# Post-Arrival Health Screening in Karen Refugees in Australia

**DOI:** 10.1371/journal.pone.0038194

**Published:** 2012-05-31

**Authors:** Georgia A. Paxton, Katrina J. Sangster, Ellen L. Maxwell, Catherine R. J. McBride, Ross H. Drewe

**Affiliations:** 1 Royal Children's Hospital, Murdoch Childrens Research Institute, Melbourne, Victoria, Australia; 2 Melbourne Pathology, Melbourne, Victoria, Australia; 3 ISIS Primary Care, Melbourne, Victoria, Australia; University of Western Australia, Australia

## Abstract

**Objective:**

To document the prevalence of nutritional deficiencies, infectious diseases and susceptibility to vaccine preventable diseases in Karen refugees in Australia.

**Design:**

Retrospective audit of pathology results.

**Setting:**

Community based cohort in Melbourne over the period July 2006–October 2009.

**Participants:**

1136 Karen refugee children and adults, representing almost complete local area settlement and 48% of total Victorian Karen humanitarian intake for the time period.

**Main Outcome Measures:**

Prevalence of positive test results for refugee health screening, with breakdown by age group (<6 years, 6–11 years, 12–17 years, 18 years and older).

**Results:**

Overall prevalence figures were: anaemia 9.2%, microcytosis 19.1%, iron deficiency 13.1%, low vitamin B_12_ 1.5%, low folate 1.5%, abnormal thyroid function tests 4.4%, vitamin D<50 nmol/L 33.3%, hypocalcaemia 7.4%, raised alkaline phosphatase 5.2%, abnormal liver transaminases 16.1%, hepatitis B surface antigen positive 9.7%, hepatitis B surface antibody positive 49.5%, isolated hepatitis B core antibody positive 9.0%, hepatitis C positive 1.9%, eosinophilia 14.4%, *Schistosoma* infection 7%, *Strongyloides* infection 20.8%, malaria 0.2%, faecal parasites 43.4%. Quantiferon-gold screening was positive in 20.9%. No cases of syphilis or HIV were identified. Serological immunity to vaccine preventable diseases was 87.1% for measles, 95% for mumps and 66.4% for rubella; 56.9% of those tested had seroimmunity to all three.

**Conclusions:**

Karen refugees have high rates of nutritional deficiencies and infectious diseases and may be susceptible to vaccine preventable diseases. These data support the need for post-arrival health screening and accessible, funded catch-up immunisation.

## Introduction

Australia accepts 13,750 refugees annually under its Humanitarian program. Source countries vary from year to year, however since 2006 there have been increasing numbers of refugees from Burma (Myanmar), reflecting regional resettlement priorities. Many of these refugees are of Karen ethnicity and have arrived from refugee camps in Thailand situated along the Thai-Burma border. Large numbers of Karen people have settled in outer metropolitan Melbourne.

Karen refugees have complex medical needs and are vulnerable to poor health for multiple reasons. These include ongoing conflict and human rights violations, poor conditions and disruption of health services in their countries of origin and transit, different patterns of communicable diseases, and issues associated with migration, settlement and accessing services in a new country.

All permanent entrants to Australia undergo a visa health assessment approximately six months prior to departure and many also undergo voluntary pre-departure medical screening (PDMS) three to five days prior to travel (Table 1). A post-arrival health assessment is also recommended, to ensure the health of the individual, provide immunisation catch-up and to identify conditions of public health significance [1]. Post-arrival refugee health assessment is voluntary, and in Victoria, is performed by General Practitioners (GPs) in primary care.

**Table 1:** Visa Medical Examination and Pre-departure Medical Screening (PDMS) [31].

**Visa Medical Examination**	
Height and Weight	All applicants
Full medical examination	All applicants
Urine dipstick testing	Applicants ≥5 years
Chest x-ray (CXR)	Aged ≥11 years and younger if any indications of TB disease or a history of contact with TB
Human Immunodeficiency Virus (HIV) serology	All applicants aged ≥15 years
Syphilis serology	All applicants aged ≥15 years
Hepatitis B virus (HBV) screening	International adoptees, unaccompanied minors, pregnant women
**PDMS – full assessment** *	
Fitness to fly assessment	All applicants
RDT for malaria and treatment if positive	All applicants
Albendazole dose given	All applicants ≥6months
MMR vaccination given	9 months–30 years of age

* There is variation in the timing of the visa medical examination, and uptake of PDMS and screening may be limited, especially for children. The full PDMS is used in Thailand (the port of departure for most of this cohort). A short form of PDMS is also used in some ports of departure, which is an assessment of ‘fitness to fly’.

Recent Australian guidelines for refugee health screening were developed for people from African source countries [1]–[3]. Data are lacking on the prevalence of health issues for people from more recent Humanitarian source countries, such as Burma. It is important to ascertain health issues for new Humanitarian arrivals in order to respond appropriately to their health care needs, evaluate practice guidelines and provide responsive, evidence-based health care.

Our aim was to document the prevalence of nutritional deficiencies, infectious diseases and susceptibility to vaccine preventable diseases in a community-based cohort of Karen refugees in Melbourne.

## Methods

The study was approved from the Human Research Ethics Committee of the Royal Children's Hospital (RCH), and received a waiver of informed consent from the institution due to the retrospective collection of de-identified data. Approval was also obtained by the Board of Management of the Community Health Centre (CHC) and Executive of the Pathology provider prior to commencement, as these institutions did not have specific Ethics committees.

A retrospective audit was performed on the pathology screening results for a cohort of Karen refugees settling in outer metropolitan Melbourne from July 2006–October 2009. During this period, all new Humanitarian entrants were referred by the local settlement support agency to a single CHC for post-arrival health screening, within two weeks of arrival. The vast majority originated from Mae La refugee camp on the Thai-Burma border and had undergone full-PDMS. Refugee health assessments and investigations (Table 2) were performed by two GPs, based on Victorian and Australian guidelines [1], [2]. A single local commercial pathology provider performed investigations. During the early stages of the study period, screening tests were not completed uniformly; due to pressures on service delivery with a large influx of refugees, provider discretion in screening, and challenges with implementing primary care refugee screening. Screening guidelines were applied with greater consistency after consolidating protocols with the pathology provider in November 2007.

**Table 2:** Investigations.

Recommended investigations [1]–[3]	Additional investigations*
Full blood examination (FBE)	Calcium
Iron studies	Phosphate
Liver function tests (LFT)	Vitamin B12
25-hydroxy vitamin D (25(OH)D)	Folate
Vitamin A (children)	Vitamin A (all ages)
Hepatitis B serology (surface antigen (sAg), core antibody (cAb), surface antibody (sAb))	Thyroid function tests (TFT)
Hepatitis C antibodies	Measles serology
HIV screening	Mumps serology
*Strongyloides* serology	Rubella serology (adolescents, adults)
*Schistosoma* serology	Hepatitis A serology
*Treponema pallidum* haemagglutination (TPHA)	
Malaria thick/thin films and immunochromatographic (ICT) testing	
Quantiferon-gold®	
Urine *Chlamydia* DNA	
Fresh faecal specimen	

* Done routinely at the Community Health Centre at the time.

Participants were identified from the pathology provider database using a combination of refugee screening identifiers (*Schistosoma* serology, ordering GP and CHC location) and taking the first test ordered of each of the pre-defined set of refugee screening investigations. Individual pathology identification numbers and names were crosschecked against the electronic data system of the CHC to verify ethnicity using naming convention and language (Karen language), which was recorded in the individual case notes.

Data were checked for duplicates and missing information, then de-identified for analysis and manipulated using Microsoft Excel (Microsoft Corporation, Redmond, Washington). Age and gender specific reference ranges used by the pathology provider were used to define normal results, except for vitamin D, where mild deficiency was defined as <50 nmol/L and moderate – severe deficiency as levels ≤25 nmol/L [4], [5].

Results are presented by age groups in order to appraise health issues across the lifespan and to inform both adult and paediatric health care. Fisher's exact test was used to compare proportions where appropriate (GraphPad Software Inc).

The Department of Immigration and Citizenship (DIAC) Settlement Reporting Facility (SRF) [6] was checked for numbers of Humanitarian entrants of Karen ethnicity settling in the Local Government Area (LGA) over the study time period.

## Results

In total 1190 people were identified from the Pathology provider database using the combination of refugee identifiers, 1136 (95%) were Karen ethnicity (49% female), and were included in the study. The Karen cohort ranged in age from 6 months to 87 years (Table 3). The DIAC SRF identified 1039 Karen arrivals (49% female) to the local area over the same time period [6].

**Table 3:** Age and gender of Karen refugee cohort.

Age	<6 years	6–11 years	12–17 years	≥18 years	Total
**Female**	61	96	92	301	550
**Male**	73	106	75	319	573
**Not recorded**	-	-	1	12	13
**Total**	134(12%)	202(18%)	168(15%)	632(55%)	1136(100%)

Screening results are shown in Table 4. Screening for haematology/nutrition parameters, thyroid function, Tuberculosis (TB), *Schistosoma*, *Strongyloides*, faecal pathogens and STIs was performed throughout the study period. Screening for malaria was completed on 427 consecutive Karen patients over the period July 2006–July 2007, and was then only used on a discretionary basis for those with symptoms. Screening for seroimmunity to measles, and mumps commenced in February 2007 (958 arrivals after this time), screening for corrected calcium levels commenced in August 2007 (609 arrivals after this time), routine screening results for hepatitis B, C, A and HIV were available from November 2007 (544 arrivals after this time); screening for rubella immunity in older adolescents and adults also commenced at this time.

**Table 4:** Prevalence of health issues by age group.

Parameter*	Age				
Haematology and nutrition	<6 years	6–11 years	12–17 years	18 years +	Overall
Low haemoglobin	23.1%(30/130)	11.6% (23/199)	3.0% (5/166)	7.1% (44/618)	9.2% (102/1113)
Microcytosis	36.9%(48/130)	23.6% (47/199)	15% (25/167)	15.1% (95/630)	19.1% 215/1126
Macrocytosis	0% (0/130)	0% (0/199)	0% (0/167)	0.2% (1/630)	0.1% (1/1126)
Neutropaenia	0.8% (1/130)	0% (0/199)	1.2% (2/167)	1.4% (9/630)	1.1% (12/1126)
Eosinophilia	9.3% (12/129)	9.0% (18/199)	21.0%(35/167)	15.4% (97/630)	14.4%(162/1125)
Low ferritin	30.2%(39/129)	8.0% (16/201)	10.2%(17/167)	11.9% (75/628)	13.1%(147/1125)
Low vitamin B12	1.9% (2/106)	0.7% (1/135)	0.9% (1/111)	1.7% (8/460)	1.5% (12/812)
Low folate	1.7% (2/121)	0.5% (1/193)	0.6% (1/166)	2.0% (12/614)	1.5% (16/1094)
Low vitamin A	2.6% (3/114)	3.1% (5/160)	0% (0/132)	0.4% (2/515)	1.1% (10/921)
Vitamin D<50 nmol/L	30.0%(39/130)	33.3%(67/201)	44.3%(74/167)	31.1%(195/627)	33.3%(375/1125)
Vitamin D≤25 nmol/L	3.1% (4/130)	2.5% (5/201)	7.2% (12/167)	2.9% (18/627)	3.5% (39/1125)
High ALP	4.6% (6/130)	4.5% (9/201)	4.8% (8/167)	5.7% (36/631)	5.2% (59/1129)
Hypocalcaemia	7.4% (5/68)	7.8% (6/77)	17.9% (14/78)	5.0% (17/343)	7.4% (42/566)
**Other**					
Low TSH	1.6% (2/125)	0% (0/197)	3.6% (6/168)	4.9% (30/618)	3.4% (38/1108)
High TSH	0.8% (1/125)	0% (0/197)	0.6% (1/168)	1.5% (9/618)	1.0% (11/1108)
High ALT	1.5% (2/130)	4.0% (8/201)	6.0% (10/167)	25.7%(162/631)	16.1%(182/1129)
**Infectious diseases**					
Hepatitis B sAg positive	0% (0/78)	4.6% (4/87)	10.3% (8/78)	13.4% (41/305)	9.7% (53/548)
Hepatitis B cAb positive	6.7% (5/75)	24.1% (21/87)	53.8% (42/78)	74.8%(228/305)	54.3% (296/545)
Hepatitis C positive	0% (0/69)	0% (0/74)	0% (0/71)	3.3% (10/305)	1.9% (10/519)
*Schistosoma* serology positive†	3.0% (4/134)	5.4% (11/202)	4.8% (8/168)	9.0% (57/632)	7.0% (80/1136)
*Strongyloides* serology positive	7.1% (9/127)	9.3% (16/172)	19.0%(26/137)	28.1%(151/537)	20.8%(202/973)
Malaria ICT positive	0% (0/40)	2.1% (2/97)	1.4% (1/70)	0.4% (1/238)	0.9% (4/445)
Malaria film positive	0% (0/40)	1.0% (1/97)	0% (0/70)	0% (0/238)	0.2% (1/445)
Faecal specimen – any cysts/ova/parasites	39.8%(49/123)	58.0%(112/193)	49.7%(81/163)	37.2%(204/548)	43.4%(446/1027)
Faecal pathogens‡	26.8%(33/123)	29.5%(57/136)	19.6%(32/131)	9.7%(53/495)	17.0%(175/1027)
Urine *Chlamydia* DNA	-	-	1.8% (2/109)	0% (0/530)	0.3% (2/649)
Quantiferon-gold®-positive	5.3% (1/19)	21.8% (12/55)	14.9%(20/134)	22.6%(136/602)	20.9%(169/810)
**Vaccine preventable diseases**					
Measles IgG positive	97.6% (41/42)	87.5% (63/72)	82.5%(94/114)	87.3%(446/511)	87.1%(644/739)
Mumps IgG positive	78.6% (33/42)	94.4%(68/72)	97.4%(111/114)	95.9%(489/510)	95.0%(701/738)
Rubella IgG positive	-	-	84.7% (50/59)	62.3%(188/302)	66.4%(245/369)
Hepatitis B sAb positive	69.7%(53/76)	39.1% (34/87)	43.6%(34/78)	48.9%(149/305)	49.5%(270/546)
Hepatitis B cAb & sAb positiveŞ	1.9% (1/53)	38.2% (13/34)	85.3% (29/34)	87.2(130/149)	64.1%(173//270)
Hepatitis A IgG positive	22.7% (17/75)	83.9% (73/87)	89.6% (69/77)	98.9%(274/277)	83.9%(433/516)

* Low and high defined by reference range for age/gender. Although a proportion of the population may have results outside a reference range if it is defined according to a normal distribution; and reference ranges will not be specific to this group; in practice, most results outside the reported reference range require at least clinical assessment, and may require treatment, hence the results are reported using the reference ranges of the pathology provider.

† Defined by titre ≥1: 32.

‡ Faecal pathogens were any one of *Giardia intestinalis*, *Entamoeba histolytica/dispar*, *Cyclospora cayetanensis*, Hookworm, *Trichuris trichiuria*, *Taenia spp*, *Strongyloides stercoralis*, *Enterobius vermicularis*, *Ascaris lumbricoides*.

Ş Defined by being Hepatitis B cAb positive where sAb was detected.

Anaemia, microcytosis and iron deficiency were common across the lifespan, but were most prevalent in children <6 years. Where microcytosis was present, the proportion with low ferritin was 58.7% (27/46) in those <6 years, 2.2% (1/46) in those aged 6–11 years, 8.0% (2/25) in those aged 12–18 years and 21.3% (20/95) in adults.

A high prevalence of vitamin D deficiency (<50 nmol/L) was found across all age groups, particularly in adolescents (44.3%). Males were significantly more likely to have vitamin D≥50 nmol/L compared to females in the adolescent (50/74 vs 42/92, p = 0.007) and adult age groups (250/317 vs 177/298, p<0.0001). The proportion of people with low vitamin D levels was similar over autumn, summer and winter (29.3–32.3%) and significantly higher in spring compared to summer (40.3%, 85/211 vs 49/167, p = 0.03). Hypocalcaemia was common; particularly in adolescents, although the proportion with an elevated ALP was similar in all age groups (4.5–5.7%). In those with moderate to severe vitamin D deficiency, 10.5% (4/38) had an elevated ALP, compared to 3.9% (13/333) of those with mild deficiency and 5.5% (41/748) of those with normal vitamin D levels. No cases of clinical rickets or fractures were identified.

An age gradient was found for the presence of hepatitis B infection (both sAg and cAb). Positive Hepatitis B cAb was an isolated finding (sAg negative, sAb negative) in 9.0% (49/545) of those tested. In those who were sAg positive, hepatitis B e antigen (eAg) was also positive in 50% (4/8) of those aged 12–17 years and 26.8% (11/41) of adults. Hepatitis B infection (sAg positive) was found in four of the 10 adults with HCV infection, and co-infection with hepatitis B and *Schistosoma* was found in 2.7% (15/548) of those screened for both conditions. Raised liver transaminases were common in adults; using an upper limit of 40 IU/L, 25.7% of adults (162/631) had a raised alanine transaminase (ALT); this proportion was 56.0% (23/41) in adults who were HBsAg positive.

Vaccination accounted for nearly all hepatitis B seroimmunity (protective titres defined as ≥10 IU/L) in the youngest age group and the majority of seroimmunity in those aged 6–11 years. In adolescents and adults, over 80% of those with seroimmunity had been exposed to HBV and were cAb positive/sAb positive.

There was a single case of malaria detected on blood film. There were no cases of active TB disease, no cases of HIV (0/541; 236 aged <18 years), no positive screening results for syphilis or gonorrhoea, and only 2 cases of *Chlamydia*.

The prevalence of pathogenic faecal parasites was higher in the younger age groups; *Giardia* was the most frequent pathogenic parasite in all age groups, and was found in 22.0% of those <6 years, 26.4% of those aged 6–11 years, 15.3% of those aged 12–17 years and 5.8% of adults. Helminth infections were found in 3.4% (35/1027), most commonly *Ascaris lumbricoides* (13 cases) and *Trichuris trichiuria* (13 cases). Multiple faecal pathogens were found in 2.9% (13/1027). Overall 78.5% (803/1027) of (fresh) faecal specimens were noted to be unformed.

Seroimmunity to vaccine preventable diseases varied. Rubella seroimmunity (defined by titre ≥28 IU/L) was 84.7% (50/59) in those aged 12–17 years and 62.3% (188/302) in adults. Of 367 people tested for seroimmunity to all three components of measles, mumps and rubella, 56.9% (209/367) had protective antibody titres.

## Discussion

This study represents the first community-based prevalence data in Karen refugees in Australia and the largest cohort of Karen refugees in the Australian literature.

Nutritional deficiencies and infectious diseases were common in Karen refugees. Anaemia and microcytosis were common, particularly in young children, where iron deficiency was prominent. Haemoglobinopathies [7] and lead toxicity [8], [9] are further possible causes/contributors to microcytosis in Karen refugee children, although testing for these conditions is not part of current Australian screening protocols.

Vitamin D deficiency is now well described in African refugee cohorts in Australia, and is reported in over 85% of African children [10], and adults [11] in Melbourne. An unexpectedly high prevalence of vitamin D deficiency was found in Karen refugees, particularly in females, and hypocalcaemia was common. Despite these findings, the proportion with an elevated ALP (suggesting increased bone turnover) was relatively constant in those with low vitamin D (compared to those with normal levels) and in the different age groups, without an increase in the youngest and adolescent age groups, as might be expected during periods of rapid growth. Most people in the Karen community are Fitzpatrick skin type III–IV, do not wear covering clothing, and in this cohort, had recently arrived from areas with high incident sunlight exposure. There were no other obvious contributors to low vitamin D levels; obesity or use of medications affecting vitamin D metabolism were both extremely uncommon. Although dietary intake of calcium was typically low, and hypocalcaemia can drive conversion of 25-OHD to active 1,25-(OH)_2_D via parathyroid hormone (PTH), a raised ALP would be expected in response to elevated PTH and an increase in 1,25-(OH)_2_D. Higher levels of 24, 25-hydroxylase (which degrades both 25-OHD and 1,25-(OH)_2_D) are reported in South Asians [12], which is another possible mechanism for reduced 25-OHD levels in this group. Low vitamin D has adverse effects on bone health, and there is an emerging body of evidence that it is associated with other effects on health [13]. Current Australian refugee screening protocols were developed in response to a wave of African migration, and include vitamin D screening based on the risk factors of this group. Given the high prevalence of vitamin D deficiency in Karen refugees, it is difficult to cease vitamin D screening at this point, although it will be important to establish whether low vitamin D remains a problem for Karen refugees after settlement, and to monitor for adverse effects on health.

Hepatitis B virus (HBV) infection was common, with an overall prevalence of 9.7%, and an age-related gradient, reaching a prevalence of 13.7% in adults. These figures compare to prevalence estimates of 0.5–0.9% for Australia [14], and 6–16% [15]–[17] in African refugee cohorts in Australia. Younger children were nearly all immune due to vaccination, likely reflecting camp immunisation programs, whereas in adolescents and adults, immunity generally reflected exposure to infection. There was a relatively high number of people with isolated positive cAb serology, which requires additional follow-up. Isolated cAb positive may represent the window phase of acute hepatitis B, a false positive result (more common in people from low prevalence areas), or, more commonly in people from high prevalence areas, immunity after previous infection with waning HBsAb, or chronic HBV infection [18].

Hepatitis B population prevalence is classified as low (<2%), intermediate (2–7%) and high (≥8%) [19]; high prevalence is usually associated with sAb prevalence of 60–80%. In this cohort, although the prevalence of sAg positive was high, over half the cohort was sAb negative, and remained susceptible to HBV. This is important from a public health perspective, as the combination of high disease prevalence and lower than expected seroprotection will facilitate disease transmission. Horizontal transmission is of particular concern within households and in young children, and is reported in other Asian refugee cohorts after settlement [20]. Household composition is often fluid in the early stages of refugee settlement, and many families stay in shared accommodation. In Australia, Hepatitis B immunisation is funded during early childhood, for sexual partners and household contacts of people with hepatitis B, and there is a catch-up program for year seven students that will cease in 2012. Hepatitis B vaccine is not funded for refugees except by these criteria. It may be prudent to assume household contact is the norm rather than the exception, and to extend funding in order to prevent transmission within the community.

The prevalence of malaria was low, likely due to the epidemiology of malaria in the area of origin, and well established local control programs. Although malaria is prevalent in the area, with 60–190 cases of malaria reported weekly in Mae La camp over the study period [21]; the endemicity is low, and populations do not acquire significant protective immunity, meaning infections are generally symptomatic (and treatment is available). This is in contrast to African refugee cohorts (from hyper-endemic areas) arriving in Australia prior to the introduction of pre-departure health screening; where asymptomatic parasitaemia and waning immunity post-migration were a significant concern [17], [22].

The prevalence of STIs was low, with similar findings in studies in Mae La camp [23] and Karen refugees in the United States [8]. The low rates in Mae La camp have been attributed to geographic isolation, religious faith, and camp governance, with defined consequences for pregnancy or pre-marital sexual relations (including forced marriage and restricting school access) [23].

*Schistosoma* infection has not been reported previously in Burma [24]. The prevalence of *Strongyloides* infection was higher than the prevalence seen in African refugees in Australia [15], [25], [26]. Pathogenic faecal parasites were more common in the younger age groups, and *Giardia intestinalis* was common in all age groups, despite pre-departure Albendazole, which has also been observed in other refugee cohorts [27].

One of the most important findings of this study was inadequate immunity to vaccine-preventable diseases, supporting the need for targeted and accessible immunisation in the early stages of settlement. The prevalence of serological immunity to rubella was low, despite PDMS immunisation with MMR vaccine, although no data were available on the timing of vaccination compared to the timing of health screening. Susceptibility to rubella and increased incidence of congenital rubella syndrome have previously been reported in South Asian cohorts [28], and this represents an important target for catch-up immunisation. Serology to detect existing immunity to vaccine-preventable diseases is not recommended in Australian refugee guidelines (except hepatitis B) [2], although serology for rubella immunity should be considered for women of childbearing age. This study highlights some of the issues with vaccination serology. Although the prevalence of seroimmunity to measles and mumps was relatively high, nearly half of those tested for all three vaccine components remained susceptible to at least one, and combination MMR vaccine is the only option available for catch-up. Similar issues occur for any combination vaccine. Almost all adults had seroimmunity to hepatitis A, suggesting routine screening is unnecessary, although targeted screening in those with liver pathology due to other causes such as hepatitis B or C is still recommended.

A coincidence of time and settlement patterns allowed the collection of these data in a community sample of Karen refugees, who currently comprise over one quarter of Australia's current offshore Humanitarian program intake. Only one other Australian study provides information on health issues in Burmese refugees [29], although this is a cohort of 156 adults referred to a specialist refugee clinic after initial health screening, representing significant referral bias. Three other studies report on smaller cohorts (31–113 people) of refugees from a mixture of Asian source countries, without breakdown by country of origin [17], [26], [30]. In the international literature, only two studies are identified on the prevalence of health issues in refugees from Burma, a small study from Canada [7] and a larger Karen cohort from Minnesota [8]. In the cohort of 1728 Karen from Minnesota, limited data are reported, although available prevalence figures are similar, including hepatitis B sAg (10%), pathogenic intestinal parasites (15%), and no sexually transmitted infections (STI) in a smaller cohort of 170 adult patients. The current study expands this literature and provides an overview of health issues affecting Karen refugees in Australia, relevant for policy makers and clinicians working in refugee health. The findings support using most tests included in current Australian refugee protocols for screening in Karen refugees, with the exception of malaria and STI testing, where targeted screening is not clinically unreasonable.

Limitations of this study include its retrospective nature, incomplete screening leading to selection bias, and possible ascertainment bias. The figures in this study reflect real life practice, and the difficulty in ordering and obtaining multiple test specimens in primary care in a complex patient group. Screening tests were not applied to all patients in a systematic manner. Protocols and testing arrangements varied over time, leading to an apparent reduction in coverage, although tests were applied to consecutive primary care arrivals. There is also the potential for ascertainment bias arising from the use of *Schistosoma* serology as an identifier (refugees not having this screening test would be missed), however this test was part of refugee health screening throughout the study period. Further, clinic attendance was voluntary, however the unusual situation of a single streamlined referral pathway to a single local CHC, with a mandated referral process, and the high attendance and follow-up seen in this cohort, led to high rates of screening uptake. The number of Karen refugees who underwent screening (1136) was slightly higher than the number settling in the local Government area over the study period (1039) [6]. This discrepancy most likely represents secondary migration, but these figures suggest that this study is likely to have captured the vast majority of new Karen refugee arrivals to the area.

Finally, it is important to note, these data are cross-sectional and do not provide information on the longer term health issues in this group, or health issues arising as a result of settlement. Initial refugee health screening is essentially a form of preventative health care to prevent complications from parasite infections, provide treatment for latent tuberculosis infection and detect hepatitis for monitoring and treatment if necessary. There is limited longitudinal information on the health of refugee cohorts in Australia. In the CHC where this study was located, obesity and type 2 Diabetes Mellitus are emerging health issues in the years after settlement.

Priorities for Australian refugee health care in the future include the need for more rapidly responsive epidemiology for new cohorts, but also developing systems to monitor longitudinal outcomes, and providing an approach to preventive health care in the initial refugee health assessment that extends beyond infectious diseases.

## References

[1] Foundation House. (2007). Promoting Refugee Health: a guide for doctors and other health care providers caring for people from refugee backgrounds. 2nd ed.

[2] Murray RJ, Davis JS, Krause V, Biggs BA, Lemoh C (2009). Diagnosis, management and prevention of infections in recently arrived refugees.

[3] Murray RJ, Davis JS, Burgner DP, Hansen-Knarhoi M, Krause V (2009). The Australasian Society for Infectious Diseases guidelines for the diagnosis, management and prevention of infections in recently arrived refugees: an abridged outline.. Med J Aust.

[4] Vitamin D and adult bone health in Australia and New Zealand: a position statement. (2005). Med J Aust.

[5] Munns C, Zacharin MR, Rodda CP, Batch JA, Morley R (2006). Prevention and treatment of infant and childhood vitamin D deficiency in Australia and New Zealand: a consensus statement.. Med J Aust.

[6] Department of Immigration and Citizenship. (2011). Settlement reporting facility.

[7] Denburg A, Rashid M, Brophy J, Curtis T, Malloy P (2007). Initial health screening results for Karen refugees: a retrospective review.. Canada Commun Dis Rep.

[8] Power DV, Moody E, Trussell K, O'Fallon A, Chute S (2010). Caring for the Karen. A newly arrived refugee group.. Minn Med.

[9] Ritchey MD, Scalia Sucosky M, Jefferies T, McCormick D, Hesting A (2011). Lead poisoning among Burmese refugee children-Indiana, 2009.. Clin Pediatr (Phila).

[10] McGillivray G, Skull SA, Davie G, Kofoed SE, Frydenberg A (2007). High prevalence of asymptomatic vitamin D and iron deficiency in East African immigrant children and adolescents living in a temperate climate.. Arch Dis Child.

[11] Skull SA, Ngeow JY, Biggs BA, Street A, Ebeling PR (2003). Vitamin D deficiency is common and unrecognized among recently arrived adult immigrants from The Horn of Africa.. Intern Med J.

[12] Awumey EM, Mitra DA, Hollis BW, Kumar R, Bell NH (1998). Vitamin D metabolism is altered in Asian Indians in the southern United States: a clinical research center study.. J Clin Endocrinol Metab.

[13] Holick MF (2007). Vitamin D deficiency.. New Engl J Med.

[14] O'Sullivan BG, Gidding HF, Law M, Kaldor JM, Gilbert GL (2004). Estimates of chronic hepatitis B virus infection in Australia, 2000.. Aust N Z J Public Health.

[15] Tiong ACD, Patel MS, Gardiner J, Ryan R, Linton KS (2006). Health issues in newly arrived African refugees attending general practice clinics in Melbourne.. Med J Aust.

[16] Johnson D (2007). Rates of infectious diseases and nutritional deficiencies in newly arrived African refugees.. http://www.sdgp.com.au/client_images/107719.pdf.

[17] Martin JA, Mak DB (2006). Changing faces: A review of infectious disease screening of refugees by the Migrant Health Unit, Western Australia in 2003 and 2004.. Med J Aust.

[18] Lok AS, McMahon BJ (2009). Chronic hepatitis B: update 2009.. Hepatology.

[19] Lavanchy D (2004). Hepatitis B virus epidemiology, disease burden, treatment, and current and emerging prevention and control measures.. J Viral Hepat.

[20] Mahoney FJ, Lawrence M, Scott C, Le Q, Lambert S (1995). Continuing risk for hepatitis B virus transmission among Southeast Asian infants in Louisiana.. Pediatrics.

[21] Shoklo Malaria Research Unit. (2011). Malaria Task Force. Mae La Camp: Total malaria cases reported weekly in 2009.

[22] Cherian S, Fagan JM, Thambiran A, Geddes J, Burgner D (2006). Severe Plasmodium falciparum malaria in refugee children despite reported predeparture antimalarial treatment.. Med J Aust.

[23] Plewes K, Lee T, Kajeechewa L, Thwin MM, Lee SJ (2008). Low seroprevalence of HIV and syphilis in pregnant women in refugee camps on the Thai-Burma border.. Int J STD AIDS.

[24] World Health Organization. (2008). International travel and health: situation as on 1 January 2008.

[25] Gibney KB, Mihrshahi S, Torresi J, Marshall C, Leder K (2009). The profile of health problems in African immigrants attending an infectious disease unit in Melbourne, Australia.. Am J Trop Med Hyg.

[26] Lucas M, Nichol P, McKinnon E, Whidboren R, Lucas A (2011). A prospective large-scale study of methods for the detection of latent *Mycobacterium tuberculosis* infection in refugee children.. Thorax.

[27] Geltman PL, Cochran J, Hedgecock C (2003). Intestinal parasites among African refugees resettled in Massachusetts and the impact of an overseas pre-departure treatment program.. Am J Trop Med Hyg.

[28] Miller E, Nicoll A, Rousseau SA, Sequeira PJ, Hambling MH (1987). Congenital rubella in babies of south Asian women in England and Wales: an excess and its causes.. Br Med J (Clin Res Ed).

[29] Chaves NJ, Gibney KB, Leder K, O'Brien DP, Marshall C (2009). Screening practices for infectious diseases among burmese refugees in Australia.. Emerg Infect Dis.

[30] Sheikh M, Pal A, Wang S, MacIntyre CR, Wood NJ (2009). The epidemiology of health conditions of newly arrived refugee children: a review of patients attending a specialist health clinic in Sydney.. J Paediatr Child Health.

[31] Department of Immigration and Citizenship. (2011). Department of Immigration and Citizenship. Form 1071i: Health requirement for permanent entry to Australia.

